# Genetic architecture of leaf morphology revealed by integrated trait module in *Catalpa bungei*

**DOI:** 10.1093/hr/uhad032

**Published:** 2023-02-21

**Authors:** Miaomiao Zhang, Bingyang Liu, Yue Fei, Xiaowei Yang, Linjiao Zhao, Chaozhong Shi, Yueying Zhang, Nan Lu, Chuangye Wu, Wenjun Ma, Junhui Wang

**Affiliations:** State Key Laboratory of Tree Genetics and Breeding, Key Laboratory of Tree Breeding and Cultivation of State Forestry Administration, Research Institute of Forestry, Chinese Academy of Forestry, Beijing 100091, China; State Key Laboratory of Tree Genetics and Breeding, Key Laboratory of Tree Breeding and Cultivation of State Forestry Administration, Research Institute of Forestry, Chinese Academy of Forestry, Beijing 100091, China; State Key Laboratory of Tree Genetics and Breeding, Key Laboratory of Tree Breeding and Cultivation of State Forestry Administration, Research Institute of Forestry, Chinese Academy of Forestry, Beijing 100091, China; State Key Laboratory of Tree Genetics and Breeding, Key Laboratory of Tree Breeding and Cultivation of State Forestry Administration, Research Institute of Forestry, Chinese Academy of Forestry, Beijing 100091, China; State Key Laboratory of Tree Genetics and Breeding, Key Laboratory of Tree Breeding and Cultivation of State Forestry Administration, Research Institute of Forestry, Chinese Academy of Forestry, Beijing 100091, China; State Key Laboratory of Tree Genetics and Breeding, Key Laboratory of Tree Breeding and Cultivation of State Forestry Administration, Research Institute of Forestry, Chinese Academy of Forestry, Beijing 100091, China; Academy of Forest and Grassland Inventory and Planning, National Forestry and Grassland Administration, Beijing 100714, China; State Key Laboratory of Tree Genetics and Breeding, Key Laboratory of Tree Breeding and Cultivation of State Forestry Administration, Research Institute of Forestry, Chinese Academy of Forestry, Beijing 100091, China; Wenxian Forestry Science Research Institute, Jiaozuo 454850, China; State Key Laboratory of Tree Genetics and Breeding, Key Laboratory of Tree Breeding and Cultivation of State Forestry Administration, Research Institute of Forestry, Chinese Academy of Forestry, Beijing 100091, China; State Key Laboratory of Tree Genetics and Breeding, Key Laboratory of Tree Breeding and Cultivation of State Forestry Administration, Research Institute of Forestry, Chinese Academy of Forestry, Beijing 100091, China

## Abstract

Leaves are crucial for maintaining plant growth and development via photosynthesis, and their function is simultaneously regulated by a suite of phenotypic traits. Although much is known about the genetic architecture of individual leaf traits, unraveling the genetic basis of complex leaf morphology remains a challenge. Based on the functional correlation and coordination of multi-traits, we divided 15 leaf morphological traits into three modules, comprising size (area, length, width, and perimeter), shape (leaf lobes, aspect ratio, circularity, rectangularity, and the relevant ratios), and color (red, green, and blue) for an ornamental tree species, *Catalpa bungei*. A total of 189 significant single-nucleotide polymorphisms were identified in the leaves of *C. bungei*: 35, 82, and 76 in the size, shape, and color modules, respectively. Four quantitative trait loci were common between the size and shape modules, which were closely related according to phenotype correlation, genetic mapping, and mRNA analysis. The color module was independent of them. Synergistic changes in the aspect ratio, leaf lobe, and circularity suggest that these traits could be the core indicators of the leaf shape module. The *LAS* and *SRK* genes, associated with leaf lobe and circularity, were found to function in plant defense mechanisms and the growth of leaves. The associations between the *SRK* and *CRK2* genes and the leaf lobe and circularity traits were further verified by RT–qPCR. Our findings demonstrate the importance of integrating multi-trait modules to characterize leaf morphology and facilitate a holistic understanding of the genetic architecture of intraspecific leaf morphology diversity.

## Introduction

Leaves are crucial for maintaining plant growth and development through photosynthesis [1]. Leaves vary tremendously in size, shape, and color within and among species, maximizing their photosynthetic efficiency by adjusting these phenotypic traits [2, 3]. Studies have overwhelmingly focused on traits related to the leaf size module (e.g. area, length, width, dry mass, and thickness), because they are easy to measure [4, 5]. Leaf shape and color, two other critical trait modules for photosynthesis, could be equally important for plant growth and receive less attention. For instance, leaf aspect ratio, circularity, and rectangularity are recognized to affect leaf photosynthetic capacity by regulating radiation absorption and gas exchange, and leaf lobes and serration characteristics are important for light foraging, as they influence light penetration of the canopy [6]. Similarly, leaf color directly reflects chemical substances, including chlorophyll, carotenoids, flavonoids, and soluble sugars, which are closely associated with photosynthetic efficiency [7]. The red/blue ratio exceeded 1.36 in nitrogen-deficient *Beta vulgaris* [8], and lighter green leaves improved the heat tolerance and water-use efficiency of *Vaccinium virgatum* [9]. Considering the importance of these phenotypic traits, the integration of these three trait modules is critical for a holistic understanding of the genetic architecture of leaf morphology and function.

Quantitative trait locus (QTL) mapping is a powerful method extensively used to identify the genetic mechanisms underlying complex traits [10, 11]. However, the reliability and precision of genetic mapping depends greatly on the accurate and high-throughput acquisition of phenotypic data. Traditional manual phenotype investigation relies on measuring and evaluating leaf traits using a ruler or vernier calipers, which is labor-intensive, time-consuming, and error-prone; manually evaluating leaf shape and color is especially problematic [12]. Advancements in imaging technology have allowed us to automatically measure leaf length and width as well as the number of leaf lobes. Image-based quantification is widely used to determine the leaf phenotype of crops [13, 14]. Despite this, interdisciplinary approaches that combine genetic mapping, advanced imaging, and molecular biology for a detailed understanding of the genetic architecture of leaf morphs is still lacking, particularly for the leaves of forest trees.

Although QTLs have been used to resolve the genetic architecture of important leaf traits, the lack of verification of the biological functions of the obtained genes means that the detected QTLs cannot be effectively used. The systems genetic strategy, which integrates QTL with other molecular biological techniques, such as transcriptional analysis, reverse transcription quantitative PCR (RT–qPCR), and transgenic experiments, is beneficial for identifying the genes underlying complex traits. This strategy has been used for photosynthesis, wood formation, and salicylic acid biosynthesis in *Populus* [15]. By combining genome-wide association studies, QTL expression mapping, co-expression analysis, and *Arabidopsis* genetic transformation assays, *PtoMYB80* was found to regulate the trade-offs between leaf area and hemicellulose content in *Populus* [16]. A combination of QTL mapping and expression pattern analysis revealed that the regulatory mechanisms of core genes are network-dependent, involve different developmental outcomes, and help determine upstream and downstream regulators [17]. However, studies on the genetic architecture of leaf growth and morphology using a system genetics strategy remain scarce.

*Catalpa bungei* C.A.Mey belongs to the Bignoniaceae family and is a valuable ornamental and timber tree species native to China [18]. The leaf morphs of *C. bungei* are mostly triangular or cordate (heart-shaped) with an acuminate or caudate leaf tip and an indented leaf blade margin. The leaf morphology varies with genotype owing to long-term evolution, and some cultivars also exhibit morphological plasticity in heterogeneous environments. In the current study, an *F*_1_ segregating population derived from *C. bungei* ‘7080’ and *Catalpa duclouxii* ‘16-PJ-3’ was established to comprehensively assess the genetic basis of leaf traits using a modular phenotypic analysis strategy. Fifteen leaf traits were obtained from 178 individuals and divided into three trait modules (leaf size, shape, and color) for genetic mapping and transcriptome analysis. We conducted QTL analysis to dissect the genetic architecture underlying leaf morphology at the module level. Candidate genes were investigated, validated, and screened by integrating QTL, transcriptional analysis, and expression network construction. Our study aims to reveal the genetic architecture of leaf growth, shape, and color formation in *C. bungei* and lays the foundation for the genetic improvement of trees.

## Results

### Variation in leaf traits and correlations

**Figure 1:**
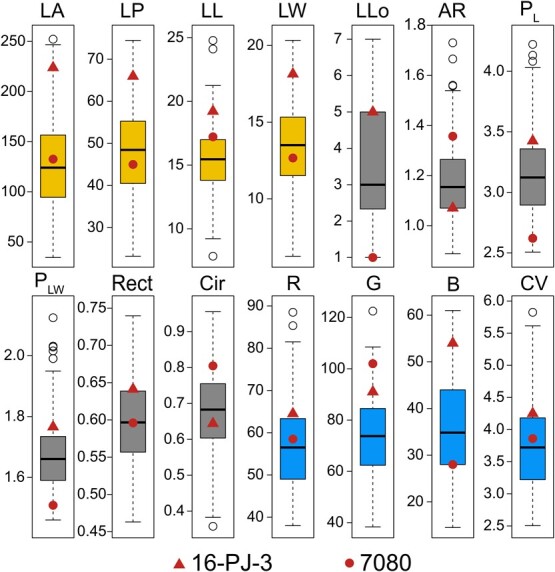
Box plot of 14 leaf traits of the *C. bungei* F_1_ population. The horizontal line represents the mean and the vertical lines mark the range from the 5th to 95th percentiles of the total data. The male parent ‘16-PJ-3’ is annotated by a triangle and the female parent ‘7080’ by the circle. Different trait modules are distinguished by color: yellow for the leaf size module, grey for the leaf shape module, and blue for the leaf color module.

A wide range of variation was observed in the 14 quantitative leaf traits. All traits showed significant differences between the parents (Fig. 1), particularly leaf area and the number of lobes. The leaf area of ‘16-PJ-3’ was 68.93% larger than that of ‘7080’, while the number of lobes of ‘16-PJ-3’ was only a fifth of that of ‘7080’ (Table 1). The coefficient of variation of the 14 traits ranged from 7.53 to 45.69% (Table 1), and can be observed intuitively in the leaf photographs (Supplementary Data Fig. S2). The number of lobes had the highest coefficient of variation, suggesting that the shape of the leaves showed abundant variation. Frequency distribution analysis (Supplementary Data Fig. S3) showed that most of the trait values were normally distributed, and can be used for further QTL analysis. The skewness and kurtosis of all the leaf traits were <2, indicating a normal distribution in the population, which was consistent with the frequency distribution analysis in Supplementary Data Fig. S2.

**Table 1:** Summary statistics of the leaf traits in the F_1_ population of *Catalpa bungei*.

**Parameter**	**LA**	**LP**	**LL**	**LW**	**LLo**	**AR**	**P**_**L**_	**P**_**LW**_	**R**	**C**	**R**	**G**	**B**	**CV (× 10** ^**6**^ **)**
Mean	128.68	48.57	15.50	13.49	3.27	1.17	3.14	1.68	0.60	0.68	57.00	74.24	35.84	3.76
Median	124.09	48.46	15.46	13.50	3.00	1.15	3.12	1.66	0.60	0.68	56.50	73.75	34.83	3.72
Std_dev	44.90	9.78	2.68	2.73	1.49	0.15	0.36	0.13	0.05	0.12	10.70	15.39	10.32	0.71
Variance	2015.66	95.57	7.18	7.45	2.23	0.02	0.13	0.02	0.00	0.01	114.56	236.72	106.45	0.51
Max	252.21	74.47	24.79	20.33	7.00	1.73	4.22	2.12	0.74	0.96	88.50	122.50	61.00	5.83
Min	34.51	23.20	7.85	6.25	1.00	0.89	2.51	1.46	0.46	0.36	38.00	38.33	14.50	2.50
Range	217.70	51.27	16.94	14.08	6.00	0.84	1.72	0.66	0.28	0.60	50.50	84.17	46.50	3.32
C.V.	34.89	20.13	17.29	20.24	45.69	13.03	11.30	7.53	8.89	17.23	18.78	20.73	28.79	18.94
CSS	0.00	0.00	0.00	0.00	0.00	0.00	0.00	0.00	0.00	0.00	0.00	0.00	0.00	0.00
R_1_	61.48	14.67	3.18	3.78	2.58	0.19	0.46	0.14	0.08	0.15	14.33	22.06	16.00	0.95
SE	3.38	0.74	0.20	0.21	0.11	0.01	0.03	0.01	0.00	0.01	0.82	1.17	0.81	0.05
Skewness	0.50	0.25	0.27	0.00	0.13	0.83	0.52	0.89	0.16	−0.22	0.51	0.21	0.22	0.49
Kurtosis	−0.24	−0.11	0.69	−0.40	−0.63	0.75	0.22	0.80	−0.65	−0.11	−0.17	−0.33	−0.69	−0.20

Std_dev, standard deviation; C.V., coefficient of variation; CSS, corrected sum or squares; R1, quartile deviation; SE, standard error of mean. For explanations of leaf trait abbreviations see Table 2.

**Table 2:** List of leaf traits measured in the *C. bungei F*_1_ population with their abbreviations, formulae, and definitions in the text and figures.

**Trait module**	**Trait**	**Formula/method**	**Definition**
Leaf size	LA	Lamina	Leaf area, cm^2^
	LP	Lamina	Leaf perimeter, cm
	LL	Lamina	Leaf length, cm
	LW	Lamina	Leaf width, cm
Leaf shape	LLo	Manual counting	Leaf lobes
	AR	$\displaystyle\frac{LL}{LW}$	Aspect ratio: ratio of major axis length to minor axis length
	P_L_	$\displaystyle\frac{LP}{LL}$	Ratio of perimeter to major axis length
	P_LW_	$\displaystyle\frac{LP}{LL+LW}$	Ratio of perimeter to the sum of major axis length and minor axis length
	Rect	$\displaystyle\frac{LA}{LL\times LW}$	Rectangularity: represents how much it fills its minimum bounding rectangle.
	Cir	$\displaystyle\frac{4\mathrm{\pi} LA}{L{P}^2}$	Circularity, also known as shape factor: difference between the leaf and a circle
Leaf color	R	WSeen	Red color value (0–255)
	G	WSeen	Green color value (0–255)
	B	WSeen	Blue color value (0–255)
	CV	65536^*^R + 256^*^G + B	Color value
	CN	WSeen	Color name, using the Royal Horticultural Society Colour Chart 2015

**Figure 2:**
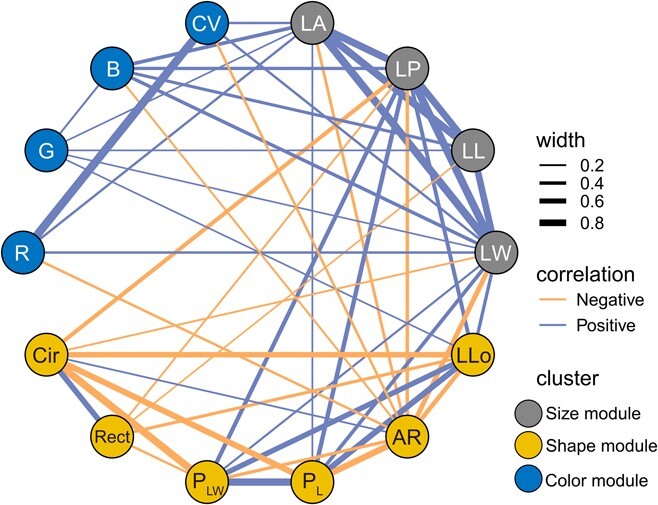
Correlation network for 14 leaf traits of *C. bungei*. Positive correlations are shown in blue and negative correlations in orange, while the strength of the correlation is represented by the width of the line. Only lines associated with *P* ≤ .05 and correlation coefficient ≥.10 or ≤−.10 are shown. Detailed correlation information for Pearson’s *r* is available in Supplementary Data Fig. S2.

Figure 2 and Supplementary Data Fig. S2 illustrate the correlation patterns among the 14 leaf traits within and between the three leaf trait modules. The leaf size traits showed strong positive correlations with each other (*r*^2^ = 0.82–0.93), which may be because the leaf area and perimeter (LP) are determined by the leaf length (LL), width (LW), and shape coefficients. The number of lobes exhibited significant correlations with all the other leaf shape traits (*r*^2^ = 0.37–0.67), which was attributed to its effect on leaf perimeter. Weak correlations were observed among the leaf color traits, except between red (R) and the color value (CV). When the leaf size and shape traits were paired, the perimeter was correlated with all the leaf shape traits (*r*^2^ = 0.22–0.50), indicating that the perimeter had the strongest effect on leaf shape among the size traits. The leaf color traits were strongly correlated with all the leaf size traits, except for the color value–leaf length pairing, suggesting that leaf color traits may affect leaf size traits through photosynthesis. However, only five of the leaf shape–leaf color pairings were significantly related.

**Figure 3:**
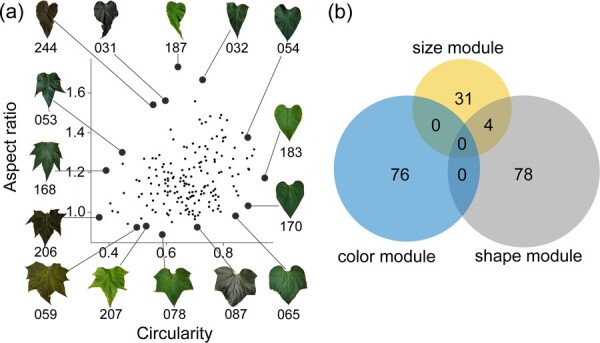
**a** Scatter plot of the leaf aspect ratio and leaf circularity of the *C. bungei F*_1_ population. Leaf photographs and names of clones with extreme aspect ratio and circularity values are provided. **b** Venn diagram of QTLs detected in the three-leaf trait module: yellow for the size module, grey for the leaf shape module, and blue for the leaf color module.

Among all the traits, the circularity, aspect ratio (AR), and number of lobes had the strongest influence on leaf shape, and the leaf shape diversity could be simply represented by these three traits (Fig. 3a). Circularity is the ratio of the area to the perimeter of the outline, which is sensitive to number of lobes and serration in the context of leaves. aspect ratio refers to the ratio of leaf length to leaf width; leaves with aspect ratio close to 1 are regarded as circular in shape, regardless of whether they possess lobes (e.g. individual 206) or not (e.g. individual 170). Abundant variation in leaf shape was observed in the scatter plots. Some individuals, such as 54, 183, and 170, had high circularity, a small number of lobes, and entire leaf margins. In contrast, some offspring (168, 206, and 59) had leaves with extremely low circularity and heavy serration, reflecting an increased perimeter relative to the blade area (Fig. 3). A high aspect ratio indicates a low number of lobes (e.g. 244, 187, and 32). For leaves with similar aspect ratio, those with more lobes showed lower circularity (e.g. 59 and 207), reflecting an increase in the perimeter relative to the leaf area.

### How quantitative trait loci govern the leaf size, shape, and color modules

**Figure 4:**
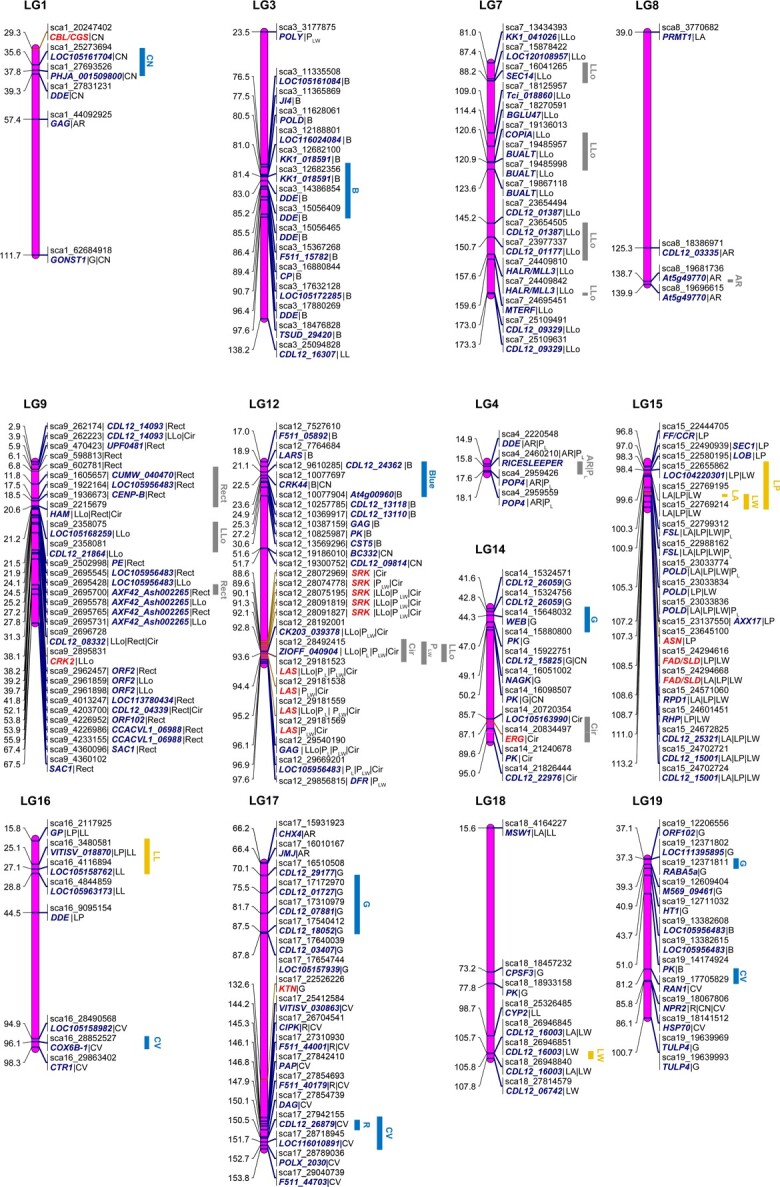
Diagrammatic genomic positions of significant QTLs detected for three leaf trait modules. The yellow bar indicates the size module, the grey bar the leaf shape module, and the blue bar the leaf color module. The genes in red were selected for in-depth analysis of gene structure, biological function and genetic effects.

A total of 189 QTLs were identified for all the leaf traits, with 118 in test crosses and 71 in inter-crosses. They were distributed on 17 LGs, mainly LG1, 9, 12, 14, 15, and 17 (Fig. 4, Supplementary Data Fig. S4; Table 3). There were 35 QTLs for the leaf size module and 16 of them were co-located. The QTLs associated with leaf perimeter, width, and area were mainly located on LG15. For the leaf shape traits, 82 QTLs were identified, 20 of which were co-located within the leaf shape module. The QTLs associated with the ratio of perimeter to the sum of major axis length and minor axis length (P_LW_), circularity, and the number of lobes were mainly located on LG12. There were 76 QTLs detected for the leaf color module. They were distributed on LG3, 12, 14, 17, and 19, with eight co-located QTLs. The colocation of QTLs explains genetic correlations between traits. There were no significant differences in the average PVE across the different modules of QTLs (size module, 7.35; shape module, 8.56; and color module, 8.43). Four co-located QTLs for the size and shape modules were associated with leaf area, perimeter, width, and length, and were located at intervals of 100.2–105.3 cM on LG15 (Fig. 3b). They explained 6.32–8.85% of the phenotypic variation. According to gene annotation, the genes in this region are thought to be related to ferruginol synthase and DNA polymerase.

The 35 QTLs for the leaf size module were distributed on six linkage groups (Fig. 4), mainly on LG15 (96.8–113.2 cM, 20 QTLs). Each QTL explained 6.01–11.66% of the phenotypic variation. The QTLs with the highest PVE values (9.34% for leaf area, 11.66% for leaf perimeter, 8.88% for leaf length, and 8.28% for leaf width) were distributed mainly on LG15 and LG16. Of the 16 co-located QTLs of the size module, 11 co-located QTLs associated with leaf perimeter and leaf width were gathered in the 98.4–113.2 cM region of LG15. The high degree of overlap between these QTLs was consistent with the significant correlation between leaf perimeter and leaf width (Fig. 2).

The 82 QTLs for the leaf shape module were distributed on 12 linkage groups, mainly on LG7 (81.0–173.3 cM, 17 QTLs), LG9 (2.9–67.5 cM, 30 QTLs), and LG12 (88.6–97.6 cM, 14 QTLs). The QTLs with the highest PVE values for each trait explained 9.99% (aspect ratio), 12.13% (circularity), 11.56% (number of lobes), 9.33% (ratio of perimeter to major axis length; P_L_), 12.15% (ratio of perimeter to the sum of major axis length and minor axis length, P_LW_), and 13.67% (rectangularity; Rect) of the phenotype variation, and were mainly distributed on LG9 and LG12. Among the 11 overlapping QTLs between LLo and circularity, eight were located in the 88.6–97.6 cM region of LG12. Four co-located QTLs for aspect ratio and P_L_ were detected in the 14.9–18.1 cM region of LG4, and showed high PVE values (7.64–9.99%).

The 76 QTLs of the leaf color module were distributed on 10 linkage groups, mainly on LG3 (76.5–97.6 cM, 14 QTLs), LG12 (17.0–30.6 cM, 10 QTLs), and LG17 (70.1–87.8 cM, 6 QTLs and 144.2–123.9 cM, 10 QTLs). Each QTL explained 5.83–14.82% of the phenotypic variation, and the highest PVE values were mainly distributed on LG3. The QTLs for the leaf color traits, including red (R), green (G), blue (B), color value, and color name (CN), could explain up to 8.69, 12.97, 14.82, 10.57, and 10.48% of the phenotypic variation, respectively. For the eight co-located QTLs for the leaf color module, five were related to R and color value, which had a close correlation with each other (Fig. 2); most of them were located in a narrow region (145.3–147.9 cM) on LG17.

### Gene ontology for leaf trait modules

Functional annotations for 116 QTLs were identified using BLAST and the National Center of Biotechnology Information ‘nr’ database. After removing duplicates, 74 annotation terms remained for all the traits. Annotation information was available for 57.14% (20), 67.07% (55) and 59.21% (45) of the size, shape, and color module QTLs, respectively. Putative genes were primarily aligned to the genomes of *Erythranthe guttata*, *Handroanthus impetiginosus*, and *Sesamum indicum* (Supplementary Data Table S2). Of these, 47 QTLs were annotated to the *H. impetiginosus* genome, with 11 in the coding sequence (CDS) gene region, 17 in the intron region, 18 in the upstream or downstream region, and 1 in the 3′-UTR region.

**Figure 5:**
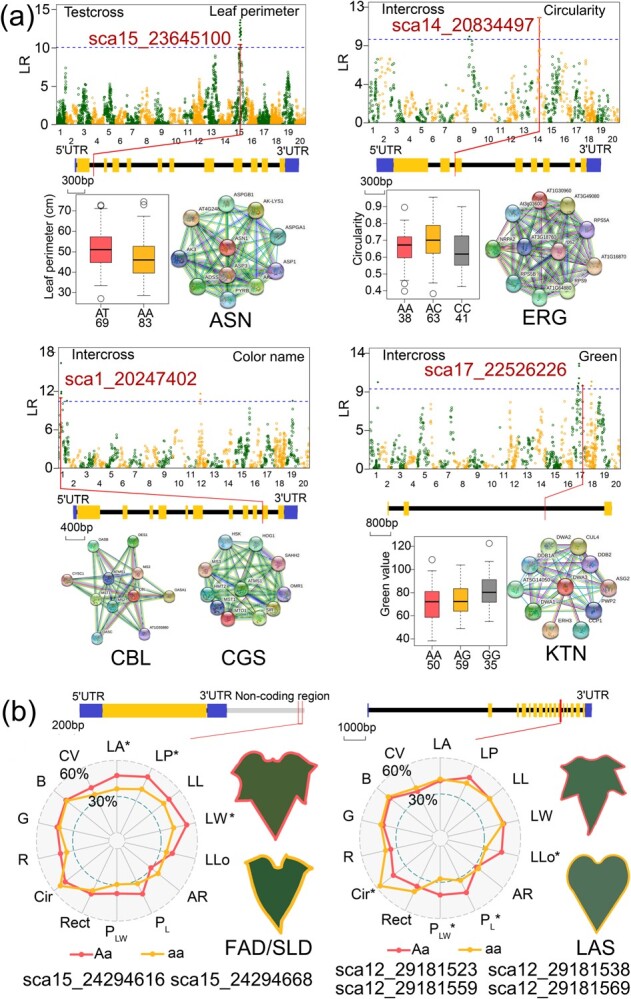
Dissecting 12 SNPs within eight important genes associated with leaf traits. **a** Manhattan plots, gene structure diagrams, protein interaction network, and genotypic box plots of the important leaf-trait-related genes, *ASN*, *ERG*, *CBL/CGS*, and *KTN*. In the Manhattan plots, the blue dashed line is the critical threshold at the 5% significance level obtained via the permutation test. For gene structure diagrams, the CDS regions inside the gene are indicated by yellow rectangles. **b** Important genes associated with multiple traits including *FAD*/*SLD* and *LAS*. Radar charts for different genotypes indicate an effect of genotypes at the peak SNP. The leaf pictures are generated by the trait mean value of different genotypes (*AA* and *Aa*).

Two important genes, *ASN* and *FAD/SLD*, were annotated in the leaf size module. QTL sca15_23645100, with a PVE of 6.84% for leaf perimeter, is located in the intron region of *ASN*, and encodes asparaginase, one of the key enzymes in plant nitrogen metabolism. It was found to regulate leaf perimeter (Fig. 5a). *ASN* interacts with *AP2* and *AP3*, encoding the aspartate kinase family protein that is involved in the first step of synthesizing the essential amino acids. From the genotypic analysis, it can be seen that leaf perimeter_AT_ > leaf perimeter_AA_. The QTLs sca15_24294616 and sca15_24294668 explained 6.25–8.40% of the phenotypic variation. Both QTLs were located on LG15 (108.5 cM), 1000 bp upstream of the *FAD/SLD* gene, which encodes delta6-fatty acid desaturase or delta8-sphingolipid desaturase. *FAD* interacts with *FaTA*, encodes the oleoyl-acyl carrier protein thioesterase 1, and plays a critical role in chain termination during *de novo* fatty acid synthesis. *SLD* interacts with ACX1, encodes peroxisomal acyl-coenzyme A oxidase 1, and may be involved in the biosynthesis of jasmonic acid. All the phenotypic values of genotype *Aa* were greater than those of genotype *aa* (Fig. 5b).

Two key genes, *LAS* and *ERG*, were predicted to regulate leaf shape. Four QTLs encode oxidosqualene-lanosterol cyclase (*LAS*), with all the SNPs located in the intron region of *LAS*, except sca12_29181569, which is located in the CDS region. *LAS* interacts with *SQE2*, *SQE3*, and *SQE6*, encodes squalene epoxidases, and catalyzes the first oxygenation step in sterol biosynthesis. The subpopulations with genotype *Aa* displayed a higher number of lobes, P_L_, and P_LW_, but lower circularity, than those with genotype *aa* (Fig. 5b). The circularity-associated QTL sca14_20834497 in the CDS region of the *ERG* gene encodes the ras-like GTPase ERA. *ERG* interacts with *RPS9* and encodes 30S ribosomal protein S9, the chloroplast ribosome component. Genotypic analysis showed that circularity_AC_ > circularity_AA_ > circularity_CC_ with circularity_AA_ and circularity_CC_ being significantly lower than circularity_AC_ (Fig. 5a).

*CBL*/*CGS* and *KTN* affected leaf color traits (Fig. 5a). QTL sca1_20247402, with a PVE of 7.07%, is located in the CDS region of the *CBL*/*CGS* gene, which encodes cyanionine beta-lyase/cyanionine gamma-synthase, the key enzyme in methionine biosynthesis. *CBL* interacts with *OASA1* and *OASB* and encodes the cytosolic isoform of cytosolic O-acetylserine (thiol) lyase, a key enzyme in cysteine biosynthesis and fixation of inorganic sulfide. *CGS* interacts with *MS2* and *MS3* and encodes 5-methyltetrahydropteroyltriglutamate-homocysteine methyltransferase, resulting in methionine formation. QTL sca17_22526226, with PVE of 7.18%, is located in the intron region of the *KTN* gene, which encodes microtubule-severing ATPase. *KTN* interacts with *DWA1* and *DWA2* and encodes DDB1-binding WD40 protein, which is hypersensitive to abscisic acid and acts as a negative regulator in abscisic acid signaling. It can be seen from the genotyping analysis that green_GG_ > green_AG_ > green_AA_.

### Gene expression pattern and candidate loci regulating leaf traits

**Figure 6:**
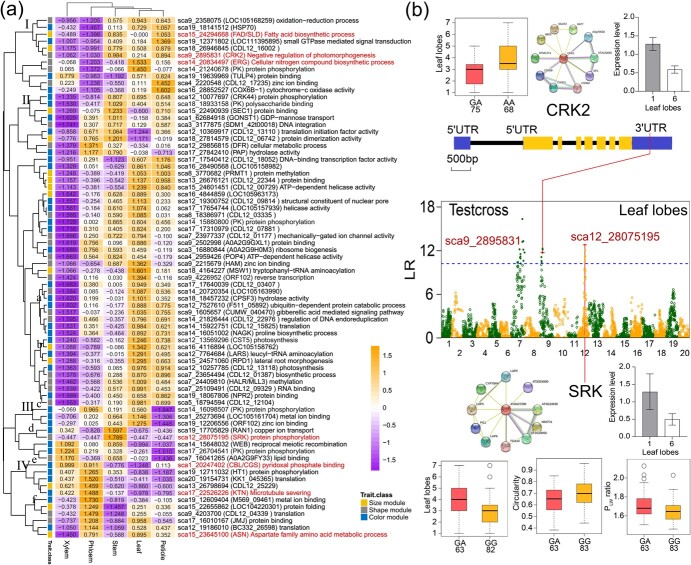
**a** Heat map of the gene expression analysis for 75 leaf-related genes using RNA-seq data from five tissues of *C. bungei*. **b** Dissecting 12 SNPs within two important genes, *CRK2* and *SRK*, associated with leaf lobe number. The corresponding SNPs are indicated by vertical red solid lines in the Manhattan plots and gene structure diagrams. The blue dashed line is the critical threshold at the 5% significance level obtained via the permutation test. CDS regions inside the gene are indicated by yellow rectangles. QTL sca_28075195 is located 40 000 bp downstream of *SRK*, so its location is not indicated. Box plots for different genotypes are plotted as an effect of genotypes at the lead SNP. The horizontal line represents the mean and the vertical lines mark the range from the 5th to 95th percentiles of the total data. Protein-protein interaction networks of homologous protein in *A. thaliana* and RT-qPCR results of *CbuCRK2* and *CbuSRK* are showed.

Using RNA-seq data from five tissues and organs of *C. bungei*, 25 466 genes were found to be expressed in the tested tissues, including 76 QTL-identified candidate genes. In general, the expression of these genes varied in the different tissues; 80% of the gene expression levels were high in the leaf, 65% of them were high in the petiole, they were relatively average in the phloem and stem, and 81% of them were low in the xylem (Fig. 6a). The expression levels of genes that regulate leaf size were almost the same in the leaves and petioles (85%); 71% were upregulated in the leaves and petioles, and 14% were downregulated. The expressed genes were enriched in many biological processes, including photosynthesis and protein phosphorylation (Supplementary Data Table S5).

Cluster analysis of gene expression values revealed three gene clusters. Most Cluster I genes were downregulated in leaf and petiole and upregulated in xylem and phloem. Cluster II contains 16 genes, most of which had low expression levels in the xylem but high expression levels in the phloem. Based on the expression levels in the stem, leaf, and petiole, Cluster II was divided into three small branches. Branch A genes were all related to leaf color; they were upregulated in the leaf and stem, but downregulated in the petiole. Cluster III included 45 genes, mainly genes related to leaf shape or leaf color. All Cluster III genes were upregulated in the leaf. Based on the level of expression in the xylem, petiole, and stem, Cluster III was divided into more branches. Seventy-five percent of members of branch A were leaf shape-related genes; their expression levels were high in petiole and xylem and low in stem and phloem. Seventy-one percent of branch B members were leaf shape-related genes, with high expression in the petiole and stem but low expression in the xylem and phloem. Seventy-one percent of the branch C members were growth-related genes and were upregulated in the petiole but downregulated in the stem, xylem, and phloem.

### *CRK2* and *SRK* regulate the formation of the leaf lobe

The seven key QTL-identified genes (Fig. 5) displayed different expression patterns in different tissues. *FAD*/*SLD* and *ERG* belonged to Cluster I, with high expression levels in the leaves, petioles, and stems, but low expression levels in the xylem and phloem. Cluster IV contained *CBL*/*CGS*, *KTN*, and *ASN*. They were upregulated in the phloem but downregulated in the stem. Compared with *CBL*/*CGS* and *KTN*, the expression levels of *ASN* were higher in the leaves and petioles but lower in the xylem. *CRK2* belonged to Cluster I. *SRK* belonged to Cluster III, with high expression levels in the stems and low expression levels in the xylem, phloem, leaf, and petiole.

RT–qPCR showed that the expression of *CbuCRK2* and *CbuSRK* was significantly different according to number of lobes, circularity, aspect ratio, P_L_, leaf area, and leaf perimeter. QTL sca9_2895831 was discovered in the 3′-UTR of *CRK2*, which encodes a cysteine-rich receptor, similar to protein kinase 2, with a large difference between the genotypes in the number of lobes (Fig. 6b). *CRK2* interacts with many genes, such as *CSN5B*, which is involved in photomorphogenesis and auxin and jasmonate responses. Based on the RT–qPCR, the expression levels of *CbuCRK2* in clones with one leaf lobe were more than twice as high as those in clones with six leaf lobes. Five QTLs were located in a narrow region of 88.6–92.1 cM in LG12. They are located downstream of the *SRK* gene, which encodes a lectin S receptor-like serine/threonine kinase. Subpopulations with the GA genotype displayed a higher number of lobes and a higher P_LW_ ratio than those with the GG genotype, and lower circularity for the GA genotype (Fig. 6b). *SRK* interacts with *CYP78A9*, a member of the cytochrome p450 family, and encodes cytochrome p450 monooxygenase. The results of RT–qPCR detection of *CbuSRK* showed the same trend as those of *CbuCRK2*.

**Table 3:** Detailed information of significant QTLs detected for the leaf traits of the *C. bungei F*_1_ population.

			**PVE**					**PVE**	
**Trait**	**SNP**	**LG (position, cM)**	(%)	**Annotation**	**Trait**	**SNP**	**LG (position, cM)**	(%)	**Annotation**
AR	sca17_15931923	17 (66.24)	7.55	CHX4	G	sca19_12206556	19 (37.15)	9.7	ORF102
AR	sca17_16010167	17 (66.37)	7.92	JMJ	G	sca19_12371811	19 (37.3)	8.29	RABA5a
AR	sca4_2460210	4 (15.76)	7.69	RICESLEEPER	G	sca19_12711032	19 (40.92)	6.51	HT1
AR	sca4_2959559	4 (18.08)	9.99	POP4	G	sca19_19639969	19 (100.72)	8.29	TULP4
B	sca12_10077697	12 (22.52)	8.97	CRK44	LA	sca15_24294668	15 (108.53)	6.25	FAD/SLD
B	sca12_10825987	12 (27.23)	9.1	PK3	LA	sca18_4164227	18 (15.57)	6.45	MSW1
B	sca12_13569296	12 (30.63)	8.39	CST5	LA	sca8_3770682	8 (38.97)	6.94	PRMT1
B	sca12_7764684	12 (18.85)	6.78	LARS	LL	sca18_25326485	18 (98.68)	6.59	CYP2
B	sca19_14174924	19 (51.01)	6.87	PK3	LL	sca18_4164227	18 (15.57)	6.81	MSW1
Cir	sca12_28072969	12 (88.64)	7.52	PK3	LLo	sca12_28075195	12 (90.08)	7.27	PK3
Cir	sca12_28074778	12 (89.64)	6.91	PK3	LLo	sca12_28091819	12 (91.29)	9.97	PK3
Cir	sca12_28075195	12 (90.08)	9.04	PK3	LLo	sca12_29181559	12 (95.2)	7.89	LAS
Cir	sca12_28091819	12 (91.29)	8.52	PK3	LLo	sca7_16041265	7 (88.17)	7.17	SEC14
Cir	sca12_29181559	12 (95.2)	12.13	LAS	LLo	sca7_18270591	7 (114.36)	10.22	BGLU47
Cir	sca14_20834497	14 (87.11)	7.82	ERG	LLo	sca7_24409810	7 (157.58)	8.59	HALR/MLL3
Cir	sca14_21240678	14 (89.55)	7.97	PK3	LLo	sca7_24695451	7 (159.64)	7.35	MTERF
Cir	sca9_2215679	9 (20.64)	6.65	HAM	LLo	sca9_2215679	9 (20.64)	6.95	HAM
CN	sca1_20247402	1 (29.26)	7.07	CBL/CGS	LLo	sca9_2895831	9 (38.1)	8.53	CRK2
CN	sca1_62684918	1 (111.7)	6.66	GONST1	LP	sca15_22490939	15 (97)	7.36	SEC1
CN	sca14_16098507	14 (50.17)	6.69	PK3	LP	sca15_23645100	15 (107.35)	6.84	ASN
CN	sca19_18067806	19 (85.82)	6.67	NPR2	LP	sca15_24294668	15 (108.53)	7.65	FAD/SLD
CV	sca16_28852527	16 (96.06)	8.94	COX6B-1	LW	sca15_24294668	15 (108.53)	6.76	FAD/SLD
CV	sca17_26704541	17 (145.33)	7.76	CIPK	Rect	sca9_2215679	9 (20.64)	8.03	HAM
CV	sca17_27842410	17 (146.77)	6.92	PAP	Rect	sca9_4226952	9 (53.81)	11.78	ORF102
CV	sca19_17705829	19 (81.23)	6.2	RAN1	Rect	sca9_4360102	9 (67.5)	7.61	SAC1
CV	sca19_18067806	19 (85.82)	8.39	NPR2	P_L_	sca12_29181559	12 (95.2)	7.54	LAS
CV	sca19_18141512	19 (86.08)	10.47	HSP70	P_L_	sca4_2460210	4 (15.76)	8.01	RICESLEEPER
G	sca1_62684918	1 (111.7)	6.88	GONST1	P_L_	sca4_2959559	4 (18.08)	7.97	POP4
G	sca14_15648032	14 (44.26)	7.34	WEB	P_LW_	sca12_28074778	12 (89.64)	7.09	PK3
G	sca14_15880800	14 (46.99)	7.02	PK3	P_LW_	sca12_28075195	12 (90.08)	8.99	PK3
G	sca14_16051002	14 (49.12)	8.43	NAGK	P_LW_	sca12_28091819	12 (91.29)	7.48	PK3
G	sca14_16098507	14 (50.17)	8.27	PK3	P_LW_	sca12_29181559	12 (95.2)	12.14	LAS
G	sca17_22526226	17 (132.57)	7.18	KTN	P_LW_	sca12_29856815	12 (97.64)	8.46	DFR
G	sca18_18457232	18 (73.24)	6.37	CPSF3	R	sca17_26704541	17 (145.33)	6.88	CIPK
G	sca18_18933158	18 (77.76)	7.74	PK3	R	sca19_18067806	19 (85.82)	7.28	NPR2

For explanations of leaf trait abbreviations see Table 2.

### Expression network construction for quantitative trait locus-identified genes

The expression networks generated for the 75 QTL-identified genes and the STRING protein–protein interaction network revealed that they are involved in a variety of biological processes, such as photosynthesis, methylation, and biosynthesis (Fig. 6). Collectively, 43 candidate genes were identified in the expression network, and were connected by 70 edges (|*r*| ≥ 0.95, *P* < .001) in *C. bungei* (Supplementary Data Fig. S5; Supplementary Data Table S6). Almost all the gene pairs (67 pairs) were significantly positively correlated. Only three pairs of genes, sca14_15648032 connected to sca14_21826444, sca14_15922751, and sca14_16051002, were significantly negatively correlated, and three of them were related to leaf color traits. Interestingly, 23 of the genes identified by QTL from the three trait modules were included in the major network, indicating that leaf morphology and color formation were the result of the interaction of multifunctional genes. Almost all the genes detected in the two smaller networks were related to the leaf color traits, implying that leaf color regulation genes work via modularization, strongly supporting the roles of the QTL-identified genes in the regulation of leaf color formation.

The hub genes, *CST5* (leaf color, sca12_13569296), *LARS* (leaf size, sca12_7764684), and *HALR/MLL3* (leaf shape, sca7_24409810), participated in chlorophyll biosynthesis, leucoyl tRNA synthase, and embryonic development. For the specific leaf color network, the key genes, *WEB* (sca14_15648032) and *NAGK* (sca14_16051002), were both related to the green value of leaf color, which is involved in abiotic stress. Subcellular localization analysis indicated that *NAGK* was targeted in the chloroplasts. The combination of QTL mapping and expression pattern analysis provides a basis for deciphering the coordinated regulatory network of genes involved in the leaf size, shape, and color modules.

## Discussion

Leaf morphology, including size, shape, and color, is key to photosynthetic carbon fixation in perennial woody plants [19]. The environmental context can affect photosynthetic rates and yield by mediating leaf morphology, such as thickness and stomatal density [20]. Natural variations in leaves are generated under natural and human selection, and a full understanding of leaf phenotypic variation and its genetic basis will be helpful for the genetic improvement of leaf shape and photosynthetic efficiency. We observed abundant leaf phenotypic diversity in the full-sib mapping population (Fig. 1) owing to the cross between *C. bungei* and *C. duclouxii*, which have distinct leaf morphologies. The leaves of the female parent ‘7080’ are smaller and pale green in color, and are long and narrow with a single-blade tip. The leaves of the male parent ‘16-PJ-3’ are larger and dark green in color, and are round with a tip containing five blades (Supplementary Data Fig. S3). This leaf phenotypic variation is consistent with other plant species, such as grapes, *Populus euphratica*, and black poplar [2, 21–23]. Despite such huge variations in leaf size, shape, and color, we still lack a systematic and logical understanding of the genetic architecture and molecular regulatory mechanisms underlying leaf morphology.

By dividing leaf traits into three modules, we found that bivariate trait relationships were generally higher within the modules than between them. The strongest positive relationships were observed within the size module, whereas the strongest negative relationships were observed within the shape module. Furthermore, there were significant correlations between the different modules of leaf traits, indicating that leaf growth and development are regulated by multiple traits. The relationship between the leaf shape and size modules was stronger than that between the leaf color and other modules, possibly because the leaf shape parameters are derived from leaf size [24]. However, the leaf shape traits are the more biologically meaningful compound traits, and specific QTLs were detected in the leaf shape module [25]. There were four co-located SNPs between the leaf shape and size modules, and the relevant QTLs interacted to form an expression regulation network for leaf phenotype formation. However, the leaf color module was independent of the other two modules, as indicated by the lack of common QTLs between the leaf color module and the other two modules. This was also evidenced by the transcriptome results, which showed that only a small expression network was composed of leaf color genes (Supplementary Data Fig. S5). Leaf color is closely related to photosynthesis in rice and wheat species [26, 27]; therefore, the leaf color and leaf growth modules were positively correlated in the current study.

Leaf circularity and aspect ratio are the two important morphometric measures for leaf shape diversity. Among the leaf traits, aspect ratio showed the strongest correlation with leaf size and color, indicating that aspect ratio, a parameter characterizing leaf shape, plays an essential role in the determination of leaf morphology. Circularity tends to covary with serration and lobe in the context of the leaves [2]. Some individuals have leaves with high circularity values, small lobes, and entire margins, whereas others have extremely low circularity values and are heavily serrated, indicating the increased perimeter compared with the blade area. For a fitted ellipse, the aspect ratio represents the ratio of the major axis to the minor axis [28]. Leaves with an aspect ratio close to 1 are considered circular, regardless of the number of lobes. Since the best-fit ellipse is used, any deviations from the circular leaf morph increases the aspect ratio and most often increases the leaf width relative to the length [2]. This suggests that aspect ratio, circularity, and serration should be comprehensively considered in subsequent studies during the selection of individuals with extreme phenotypes.

We also found several pleiotropic genes for circularity, the number of leaf lobes, and the P_LW_ ratio, especially *LAS* and *SRK*, which were identified by five QTLs. *LAS* regulates the biosynthesis of lanosterol, which is partially involved in the production of phytosterols and the synthesis of secondary metabolites in plants in response to biological and abiotic stresses. It is related to the defense mechanism of plants in response to environmental stimuli [29]. In *Solanum tuberosum*, *StLAS* is involved in the biosynthesis of phytosterols and steroidal glycoalkaloids in leaves and tubers [30]. Under *LAS* regulation, heterozygous and homozygous genotype leaves have a similar leaf color and size, whereas subpopulations with *Aa* have extremely low circularity values, high perimeters, and large lobe numbers, reflecting an increased perimeter (contributed by a large lobe) relative to the area of the blade. Therefore, we speculated that these two pleiotropic genes may be candidate genes for the regulation of leaf growth and development. *SRK* encodes a G-type lectin S receptor-like serine or threonine kinase, a member of the cell surface receptor-like protein kinase family. In *Glycine soja*, *GsSRK* was important for plant tolerance to salt stress, as evidenced by the better growth performance of the transgenic plants than that of the wild-type plants, in terms of germination rates, fresh weight, and the number of green and open leaves. Furthermore, the *GsSRK* transgenic lines had longer primary roots, higher rosette weights, and larger rosette leaves at the seedling stage [31]. Importantly, the RT–qPCR results demonstrate the influential role of *CbuSRK* in the formation of leaf traits. The individuals selected for RT–qPCR showed significant differences between the two groups, not only in leaf shape module traits, such as the number of leaf lobes, circularity, and aspect ratio, but also in leaf size traits, such as leaf area and perimeter.

Genetic mapping is a powerful method for dissecting the genetic basis underlying intricate leaf phenotypes. We identified 189 significant SNPs showing diverse phenotypic variation for 15 leaf traits in *C. bungei*, with a range of 5.83–14.82% of the PVE. This supports the opinion that leaf size and developmental properties representing complex traits are regulated by multiple loci [32]. Several regions with dense SNP loci were significantly correlated with blue (LG3, 12), green (LG14, 19), leaf serration (LG 7, 9, 12), and circularity (LG 12, 14). These SNPs provide potential molecular markers for leaf traits. Previous studies have explored the genetic architecture of leaf morphology in poplar, *Arabidopsis thaliana*, and *Hordeum vulgare* [33–35]. The leaf width on LG18 overlapped with the Q18–99 intervals reported in a previous study [18]. The QTL region of the green color (66.2–87.8 cM, LG17) overlapped with the soil and plant analyzer development value (SPAD)-related QTL region (67.2–87.8 cM, LG17) and Q17–84 reported in previous studies by Zhang *et al*. [36] and Lu *et al*. [18]. Due to the strong correlation between leaf green and SPAD, the significant green/SPAD correlation QTL region was stabilized in the annual and biennial full-sib population of *C. bungei*. The development and utilization of precise and reliable molecular markers will improve breeding efficiency. We also detected overlapping QTL regions for the leaf color module (color value and B) in LG3, the leaf shape module (circularity, P_LW_, and number of lobes) in LG12, and the leaf size module (leaf area, leaf perimeter, and leaf width) in LG15 (Fig. 4). Importantly, the co-located loci with different traits or modules provided clues for selecting improved varieties of *C. bungei*.

Genes are the fundamental cause of trait differences. Four biological processes are critical for leaf growth and development: photosynthesis, nitrogen metabolism, cell differentiation and development, and the stress response [37]. Our study annotated 74 candidate genes and several genes that were previously found to be associated with these four processes (Supplementary Data Tables S2–S4). *ASN* for the leaf size module encodes asparaginase, which is involved in nitrogen transport and storage in plants [38]. The leaf shape module *ERG* encodes the Ras-like GTPase ERA, which regulates early seed development via mitochondrial ribosome maturation and protein translation [39]. Additionally, the *CBL*/*CGS* gene for the leaf color module encoding cystathionine beta-lyase catalyzes the penultimate step in methionine biosynthesis [40]. In our study, we selected six clones with extreme phenotypic values and collected their leaves for RT–qPCR. The expression levels of *CbuCRK2* in clones with one leaf lobe were more than twice as high as those in clones with multiple leaf lobes, indicating that *CbuCRK2* negatively regulates the number of leaf lobes*.* Cysteine-rich receptor-like protein kinases, such as *CRK2* and *CRK44*, are involved in photosynthesis and are important for signal transduction [41]. *CRK2* interacts with respiratory burst oxidase homolog D (RBOHD) to control ROS production, which is the central element in coordinating the release of extracellular ROS and regulating the balance between different defense responses [42].

Unlike the *LAS* and *SRK* genes mentioned above for the leaf shape module traits, the *FAD*/*SLD* gene was co-located for all traits of the leaf size module. *FAD*/*SLD* plays a crucial role in plant growth and plant stress response and maintains the fluidity and integrity of the cell membrane by changing the synthesis and proportion of unsaturated fatty acids, thus affecting plant stress resistance [43]. *SLD* enhances the ability of plants to resist aluminum toxicity in *Populus trichocarpa* [44]. Under the effect of *FAD*/*SLD*, the leaves of heterozygous genotype *Aa* had lower circularity and aspect ratio values, a larger size (area, length, width, and perimeter), and more lobe tips than the homozygous genotype, along with a reddish coloration.

Conventional studies have focused mainly on a limited number of leaf traits or single trait-related genes that are beneficial in understanding the leaf size and development of woody plants, but lack the ability to fully decipher the interactions within and among the three leaf trait modules. By digitally extracting leaf phenotypes and using modular trait classification, QTL mapping, and expression network analysis, we dissected the genetic basis of leaf size and development in *C. bungei* and found that pleiotropic genes coordinated the three leaf trait modules. Instead of the simple single-trait genetic mapping for multidimensional phenotypic data, we propose that the 14 traits could be divided into three modules with more internal modular correlations. The color module was independent of the leaf shape and size modules, which were more closely related at the level of phenotypic correlation, QTL mapping, and mRNA analysis. Owing to the synergistic changes in the leaf aspect ratio, number of leaf lobes, and leaf circularity of *C. bungei*, these traits could be the core indicators of the leaf shape module. The genes *LAS* and *SRK*, related to the leaf lobe number and circularity, were found to be involved in the plant defense mechanisms, germination, and the growth of leaves and roots. *FAD*/*SLD*, discovered for all leaf size traits, plays a crucial role in plant growth and stress response. The leaf lobe-associated *SRK* and *CRK2* genes were further verified by RT–qPCR. Our findings demonstrate the importance of integrating multi-trait modules to characterize leaf morphology and facilitate a holistic understanding of the genetic architecture of intraspecific leaf morphology diversity. Future studies should integrate transcriptomics, metabolomics, and proteomics for multi-omics analysis of samples with extreme phenotypes to improve the precision of the elaborate regulatory networks. Furthermore, leaf growth and morphology at different growth stages (juvenile, adult, and old), and environmental factors should be explicitly considered for genetic improvement of tree breeding.

## Materials and methods

### Plant materials and single-nucleotide polymorphism genotyping

A full-sib population of 179 accessions was generated from a cross of two *Catalpa* cultivars, namely *C. bungei* ‘7080’ (female parent) and *C. duclouxii* ‘16-PJ-3’ (male parent) in 2018. Restriction-site-associated DNA library construction, sample indexing, and pooling were performed according to Baird *et al*. [45]. We called the genotypes of the 179 *F*_1_ progeny using a strict Bayesian method, and identified 9593 segregated single-nucleotide polymorphisms (SNPs). On basis of this mapping population and a set of SNP loci, a genetic map including 20 linkage groups (LGs) was constructed in a previous study. The genotyping data have been submitted to the NCBI SRA database (http://www.ncbi.nlm.nih.gov/sra) under accession number PRJNA551333 [18].

### Leaf photography and phenotype measurement

The 179 individuals and their two parents were asexually propagated and planted in the experimental field of the Luoyang Academy of Agricultural and Forestry Science, China (112.55°N, 34.71°E). A randomized block design was applied with two ramets per clone in each plot and five replicates. In September 2020, the sixth fully expanded leaf was picked from the southernmost branch in the middle and upper parts of the crown for 179 lines and the two parents, with three replicates. In total, 543 leaves were collected for subsequent photographs. The leaves were photographed against a white background using a mobile phone with a constant focal distance (Supplementary Data Fig. S1). We measured four leaf size traits (leaf length, width, area, and perimeter) using Lamina software [46]. The leaf color traits were measured using WSeen software (Hangzhou WSeen Detection Technology Co., Ltd., Hangzhou, China). The number of leaf lobes was counted manually. The abbreviations and definitions of the traits are listed in Table 2.

### Quantitative trait locus mapping

The 15 leaf traits were subjected to QTL mapping, which can be achieved by two statistical approaches: a mixing model for sparse molecular markers and a multiplicative model for dense molecular markers. Because the constructed linkage map was relatively dense, we employed a multiplicative model assuming that the QTLs were located at the positions of the markers. The multiplicative likelihood model is expressed as:

(1) \begin{equation*} L\left(\boldsymbol{\Phi} |\mathbf{y}\right)=\prod_{i=1}^{n_1}{f}_1\ \left({y}_i\right)\dots \prod_{i=1}^{n_j}{f}_j\left({y}_i\right) \end{equation*}

where **Φ** is the unknown parameter set, **y***_i_* is the vector of the leaf phenotypic values of progeny *i*, *n_j_* is the number of offspring with SNP genotype *j*, *f_j_*(*y_i_*) is the normal distribution of offspring *i* with the expected mean vector for genotype *j* (μ*_j_*), and the matrix $ \boldsymbol\Sigma $ contains the variances (σ^2^). Statistical methods based on equation (1) were used to estimate the parameters Φ = (μ*_j_*, σ^2^). The null hypothesis for the existence of the QTL is H_0_: μ*_j_* = μ, for *j* = 1 or 2. The test for the QTL effect at each position was performed by calculating the log-likelihood ratio, and the genome-wide critical threshold was determined from permutation tests.

Exploratory data analysis and visualizations, QTL mapping, phenotypic variation explained (PVE), and genetic effect calculation were performed using R version 4.0.3 [47]. The possible functions of the determined QTLs were annotated and predicted via BLAST in the National Center of Biotechnology Information ‘nr’ database (http://blast.ncbi.nlm.nih.gov/), identified on Uniprot (http://www.uniprot.org/), and analyzed for the protein–protein interaction network using STRING online protein interaction analysis software (http://string-db.org/).

### RNA sequencing and co-expression networks of candidate genes

Using the Plant RNeasy Kit (Qiagen), total RNA was extracted from various tissues (leaf, petiole, twig, xylem, and phloem tissues) of a *C. bungei* individual tree >100 years old growing in Nanyang, Henan Province, China. After quantification using NanoDrop ND-1000 and Agilent 2100 bioanalyzers, the RNA libraries were sequenced on an Illumina HiSeq 2500 platform (Illumina Inc., San Diego, CA, USA). The clean reads were assigned to the *C. bungei* reference genome using TopHat v2.0.9. Fragments per kilobase of transcript per million fragments (FPKM) were calculated by using Cufflinks v2.1.1 [48] to normalize transcript expression.

To determine the regulatory roles of the QTL-identified genes, a set of 25 466 genes that were expressed in the six *C. bungei* tissues on a genome-wide scale was used for differentially expressed gene analysis and expression network construction. FPKM values were used to generate an expression heat map for the QTL-identified candidate genes using the R package ‘pheatmap’. The network was developed based on pairwise correlations between these genes, calculated using Pearson’s correlation coefficient (*r*) in R software. A gene expression network of the QTL-identified genes was created, exhibiting gene pairs with significant correlations of |*r*| ≥ .95 and *P* < .001 using the R packages ‘tidygraph’, ‘ggraph’, and ‘ggplot2’.

### Transcriptome analyses of leaves with extreme phenotype values

To verify the effect of key genes on leaf traits, the individuals with extreme phenotype values of leaf lobe (LLo) and circularity (Cir) were selected as samples for RT–qPCR (Supplementary Data Table S1). In September 2020, 10–20 newly developed leaves were harvested from the eastern- or southernmost lateral branches, and the petioles were removed. The samples were immediately placed in liquid nitrogen and stored at −80°C for subsequent RNA extraction. Total RNA was isolated from samples by using an RNAprep Pure Plant Plus Kit (TIANGEN Biotech Co., Ltd., Beijing, China). The total RNA concentration and integrity were determined using a NanoDrop (Thermo Fisher Scientific Inc.). RNA was reverse-transcribed into cDNA using the PrimeScript™ RT Reagent Kit (Perfect Real Time; Takara Biomedical Technology Co., Ltd., Beijing, China). Primary pairs were designed for CRK2 and SRK. cDNA was amplified using a KAPA SYBR^®^ FAST Kit (Shanghai Roche Pharmaceutical Co., Ltd.), and the gene expression levels were calculated by the 2^−ΔΔCt^ method.

## Acknowledgements

The work was supported by the National Key Research and Development Plan of China (2021YFD2200202) and the National Natural Science Foundation of China (32001337). We thank Luoyang Academy of Agriculture and Forestry for providing plant materials.

## Author contributions

J.W. and M.Z. designed and supervised the research. X.Y., L.Z., C.S., and C.W. collected the phenotypic data. W.M., Y.Z., and N.L. generated and interpreted genotype data. M.Z., B.L., and Y.F. performed genetic and transcriptomic analyses, and gene expression experiments. M.Z. and B.L. drafted the manuscript. J.W. conceived the study, interpreted the results and finalized the manuscript. All authors revised the manuscript critically for intellectual content and read and approved the final version.

## Data availability

The datasets generated and/or analyzed during the current study are available from the corresponding author upon reasonable request.

## Conflict of interest

The authors confirm that they have no conflicts of interest to declare.

## Supplementary Data

Supplementary data is available at *Horticulture Research* online.

## Supplementary Material

Web_Material_uhad032
