# Supplementary figures and images for: Genetic architecture of leaf morphology revealed by integrated trait module in Catalpa bungei
Source: Hortic Res. 2023 Feb 21;10(4):uhad032. doi: 10.1093/hr/uhad032 (PMC10120837; doi:10.1093/hr/uhad032)

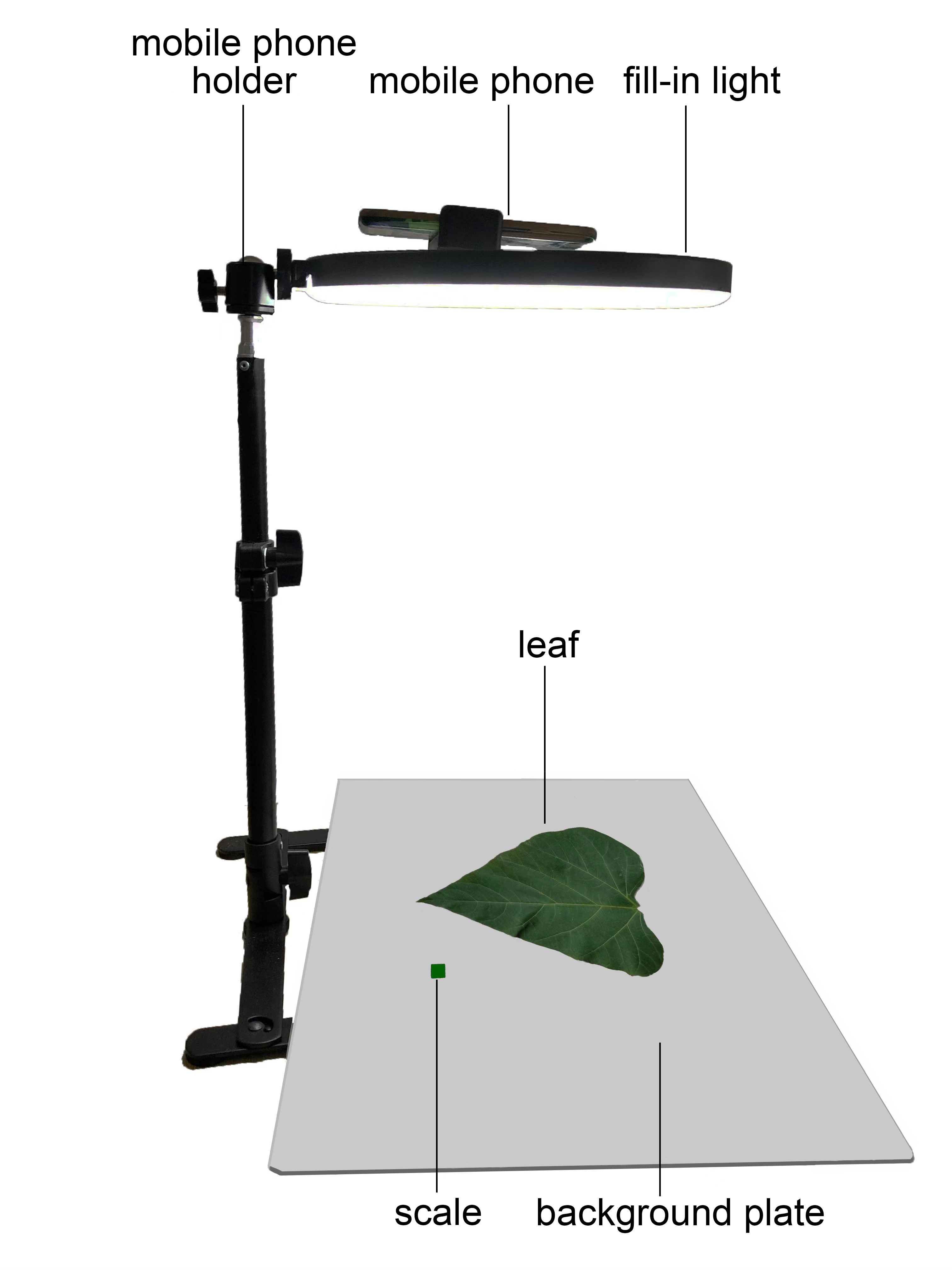

Supplement: Web_Material_uhad032 [file web_material_uhad032.zip › FigS1.Re1.tif]

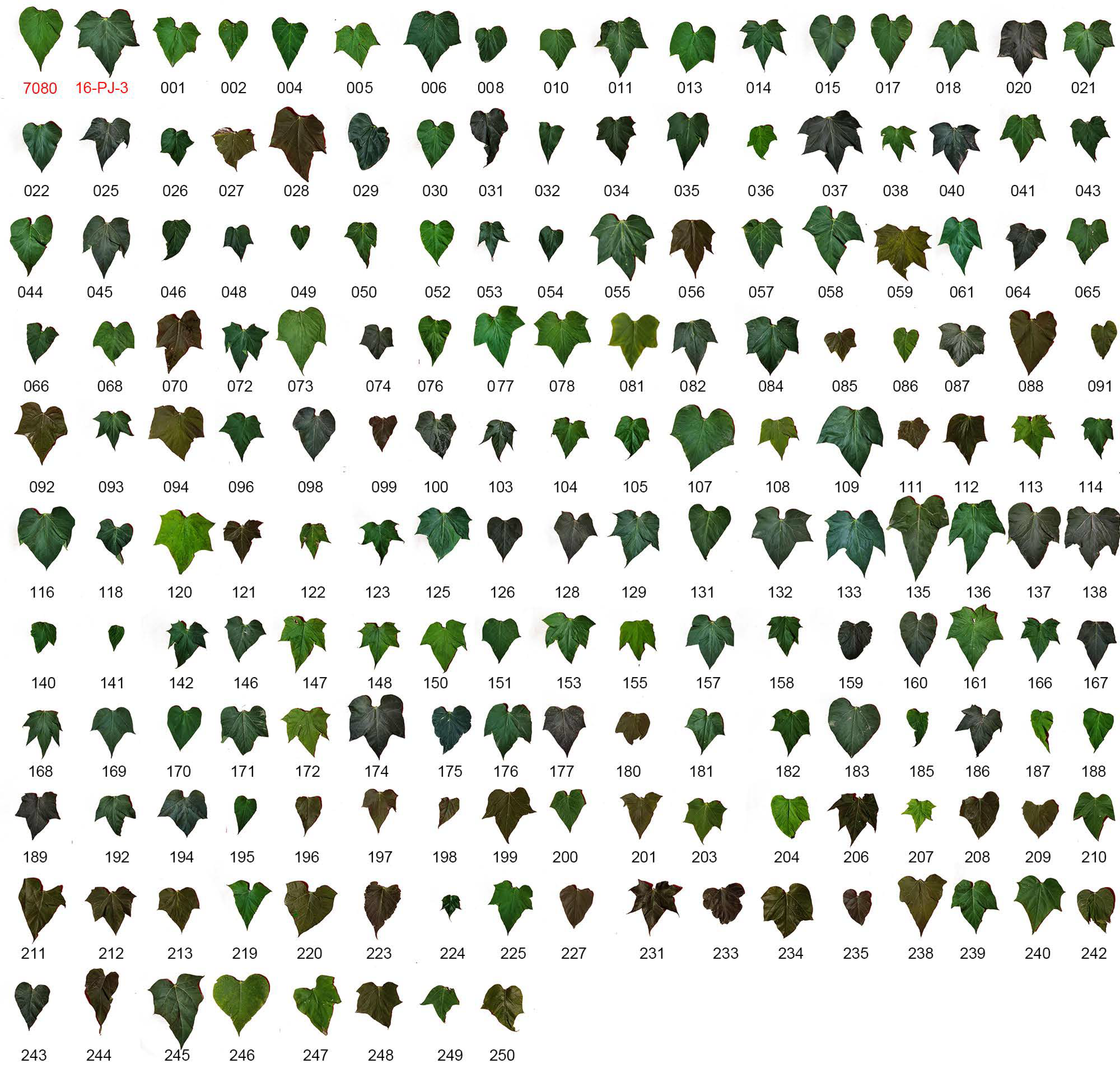

Supplement: Web_Material_uhad032 [file web_material_uhad032.zip › FigS2.Re1_01.tif]

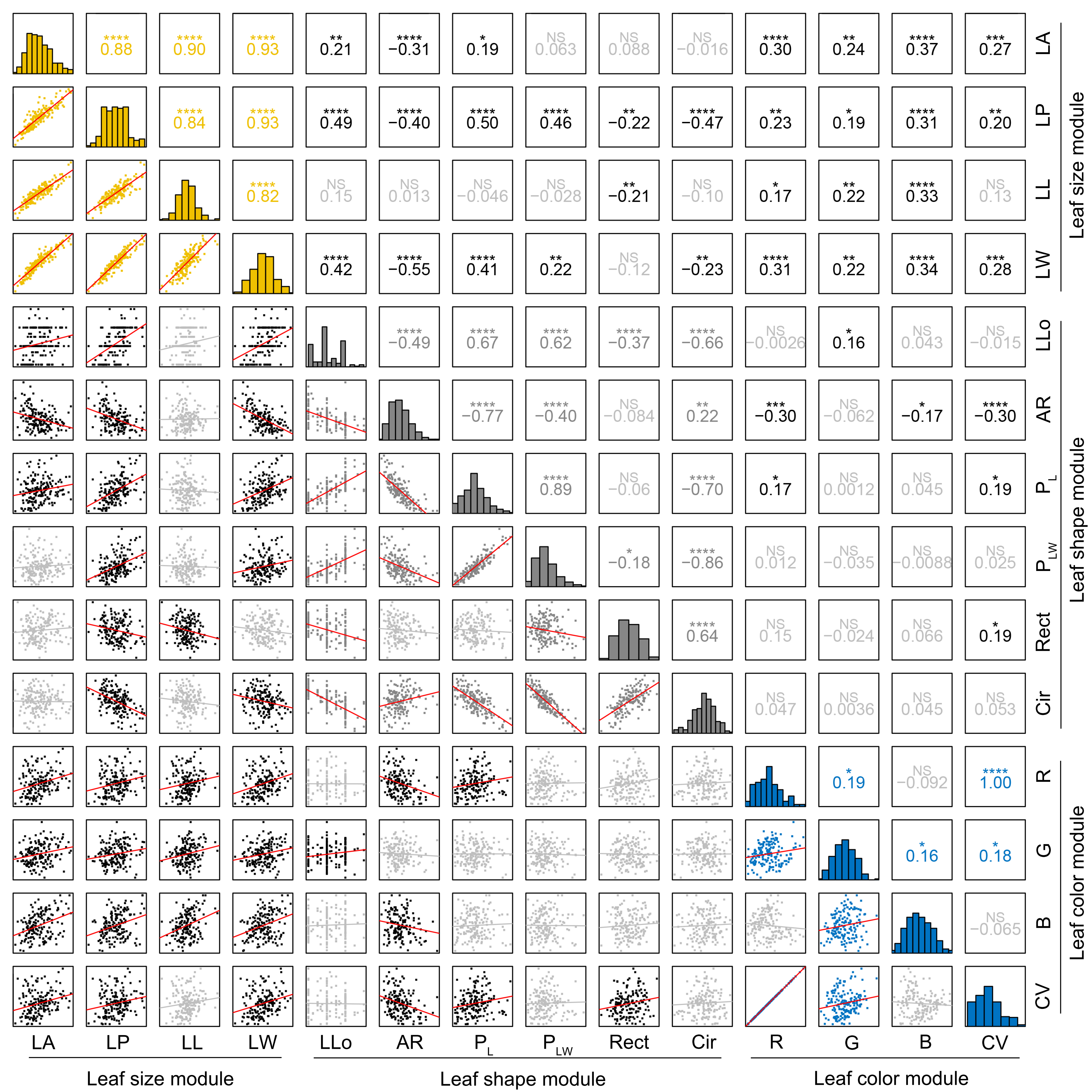

Supplement: Web_Material_uhad032 [file web_material_uhad032.zip › FigS3.Re1_01.tif]

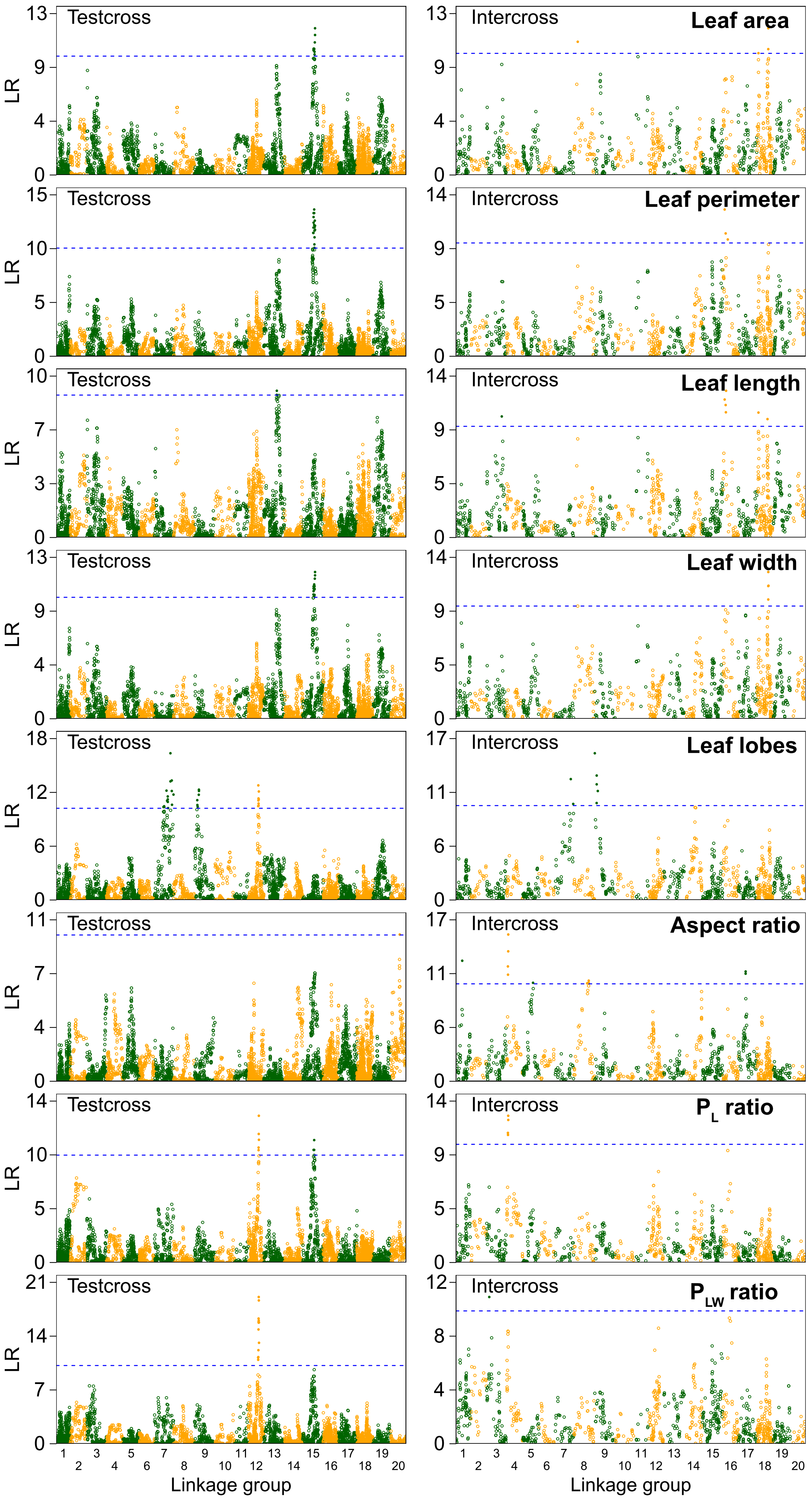

Supplement: Web_Material_uhad032 [file web_material_uhad032.zip › FigS4-1_01.tif]

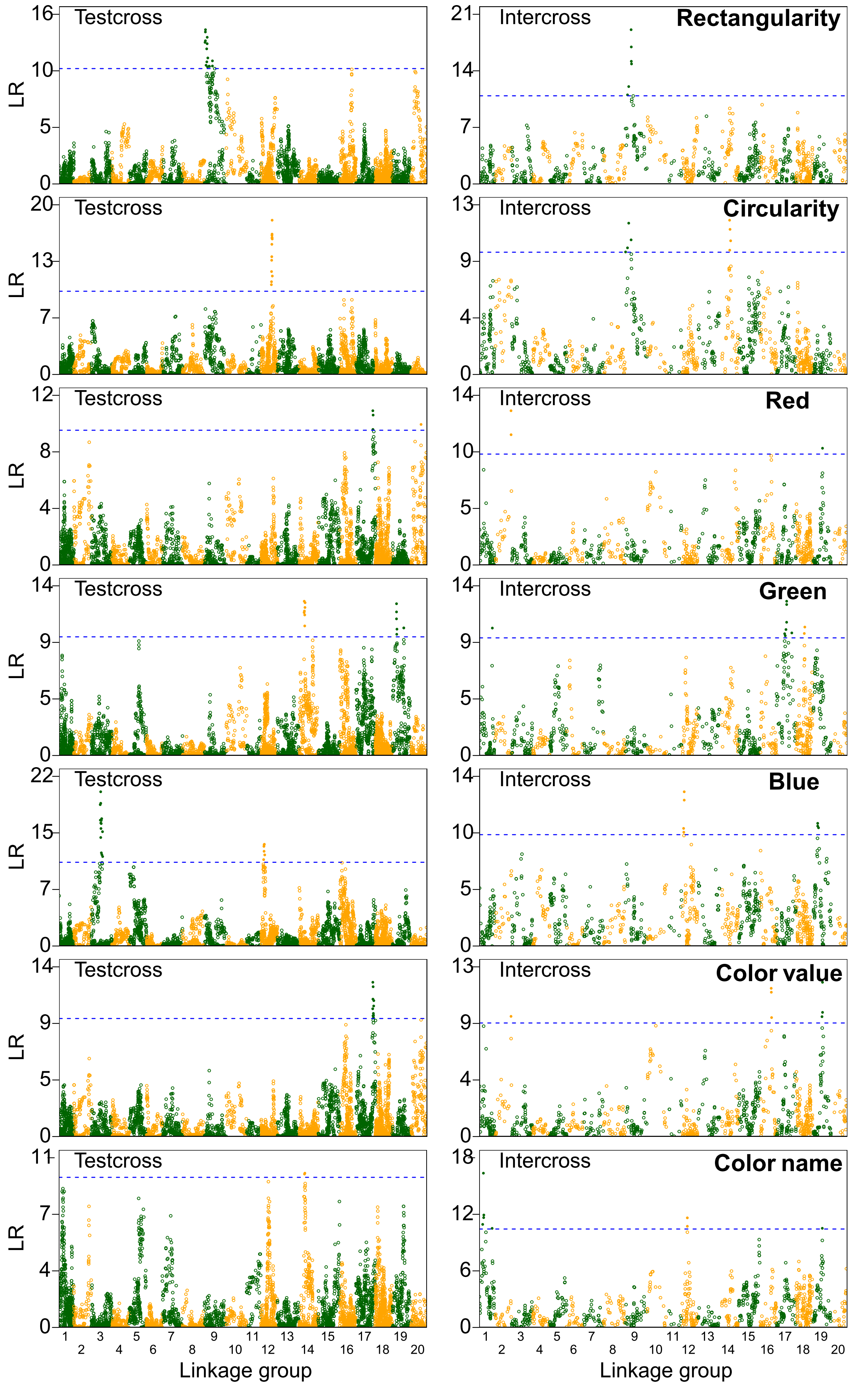

Supplement: Web_Material_uhad032 [file web_material_uhad032.zip › FigS4-2_01.tif]
